# Psychometric Evaluation of the Validity and Reliability of the Italian Version of the London Measure of Unplanned Pregnancy Amongst Postnatal Women

**DOI:** 10.3390/healthcare13162052

**Published:** 2025-08-20

**Authors:** Martina Smorti, Paul Christiansen, Geraldine Barrett, Jennifer A. Hall, Chiara Ionio, Giulia Ciuffo, Marta Landoni, Anna Maria Della Vedova, Elana Payne, Mia Richell, Semra Worrall, Giulia Mauri, Victoria Fallon, Alessandra Bramante, Sergio A. Silverio

**Affiliations:** 1 Department of Surgical, Medical and Molecular Pathology and Critical Care Medicine, University of Pisa, 56124 Pisa, Italy; 2 Department of Psychology, Institute of Population Health, University of Liverpool, Liverpool L69 7ZA, UK; 3 Research Department of Reproductive Health, Elizabeth Garrett Anderson Institute for Women’s Health, University College London, London WC1E 6AU, UK; 4 Unità di Ricerca sul Trauma, Dipartimento di Psicologia, Università Cattolica del Sacro Cuore, 20123 Milan, Italy; 5 Dipartimento di Scienze Cliniche e Sperimentali, Facoltà di Medicina e Chirurgia, Università degli Studi di Brescia, 25123 Brescia, Italy; 6 Department of Women & Children’s Health, School of Life Course & Population Sciences, King’s College London, London SE1 7EH, UK; 7 Department of Psychological Medicine, Institute of Psychiatry Psychology & Neuroscience, King’s College London, London SE5 8AF, UK; 8 Policentro Donna, 20123 Milan, Italy

**Keywords:** invariance, London measure of unplanned pregnancy, measurement, perinatal mental health, pregnancy intention, psychometrics, unplanned pregnancy

## Abstract

**Background**: Unplanned pregnancy is a public health issue and understanding women’s decision making aids practitioners in assessing fertility trends, contraception use, and family planning counselling. In Italy, Catholicism reinforces ‘natural reproduction’ and ‘traditional’ contraception, making it an ‘Imperfect Contraceptive Society.’ A valid and reliable measure of pregnancy intentionality is increasingly important, and the London Measure of Unplanned Pregnancy (LMUP) has proved effective. **Objectives and Methods**: This study comprised four stages: (1) English–Italian translation and back-translation to create the Italian version [LMUP-IT]; (2) online data collection from postnatal women; (3) evaluation of its psychometric properties (targeting, reliability, construct validity via CFA and measurement invariance with a UK sample, ‘known groups’ hypothesis testing); and (4) exploratory analysis of its associations with perinatal mental health. The sample comprised 450 postnatal women (M_age_ = 33.6 ± 4.5). **Results**: The LMUP-IT was shown to be reliable (ωT = 0.81, α = 0.76), with acceptable targeting. Measurement invariance testing confirmed consistency with the UK sample in factor structure, loadings, intercepts, and errors. LMUP-IT scores significantly correlated with well-known indicators of perinatal mental health. **Conclusions**: Overall, the LMUP-IT is a reliable measure of pregnancy intention in Italian for postpartum women. Understanding pregnancy intention will help healthcare professionals tailor interventions to better support women’s mental health during the transition to motherhood.

## 1. Introduction

Unplanned pregnancy has a significant impact on public health. It is not something which just effects low- and middle-income countries [1], with a recent survey conducted in European countries reporting nearly half of pregnancies are unplanned (the estimations range from 34% in Western to 54% in Eastern countries) [2]. A recent survey by the Italian National Statistics Institute (ISTAT) showed despite most women’s use of effective contraceptive methods, about 15% of sexually active Italian women do not use any contraceptive methods, 17% use natural methods, and 18% use the coitus interruptus, percentages higher than that reported in other European Countries [3]. Barriers to use of effective female contraceptive methods (e.g., contraceptive pill, intrauterine device, and implants) partially exist due to the mandatory medical prescription required for obtaining them, besides the lack of appropriate information on contraception, limited economic access (the contraceptive pill is not reimbursed except in specific cases, and free emergency contraception is not guaranteed in all pharmacies), the lack of comprehensive sexual education, misconceptions about risk and benefits of different birth control methods, and the presence of cultural and religious beliefs leading to moralistic attitudes [3,4]. In fact, the Catholicism typical of Italian culture [5], which emphasizes ‘natural reproduction’ and to the use ‘traditional’ contraceptive methods, leads to the definition of Italy as an ‘Imperfect Contraceptive Society’ [6].

An unplanned pregnancy may not mean an unwanted pregnancy. Rather, pregnancy planning and intention is a complex multifaceted concept including varying degrees of pregnancy intention from inappropriate timing to unwanted conceptions [7]. There are several factors which may constitute a risk for unplanned pregnancy, such as younger age, couple status (being single women or women living without a partner is a risk factor compared to being in couple married or co-habiting), lower education, and multiparity [8,9,10,11,12,13].

Planned pregnancies allow women to implement healthy behaviours in the months before conception. Thus, an unplanned pregnancy may see women fail to correct more unhealthy pre-pregnancy behaviours, which may have lasting effects on the good progress of the pregnancy and maternal well-being [14,15]. However, in some cases, the burden of an unplanned pregnancy may lead to the choice of an elective abortion, which may present mixed consequences for women and their families [16]; these are handled with great variation between societies and their respective healthcare systems. Moreover, women who choose to continue with an unplanned pregnancy tend to report significantly more mood disturbance—both during pregnancy and in the first postpartum year [17]—and in some cases increased levels of depression and anxiety to the point of being clinically relevant [18,19]. Finally, infants born from an unplanned pregnancy are at higher risk for mortality, as well as behavioural and psychological disorders [20,21].

For the above reasons, unplanned pregnancy is of considerable interest to public health professionals. Furthermore, pregnancy planning can be used to understand population fertility, the relationship with contraceptive methods, and when counselling for family planning [22]. Evaluating intentionality and the desire for pregnancy may also assist interventions to prevent the onset of unwanted pregnancies and may improve the psychological health of the woman and—where applicable—her partner as well. It is therefore imperative to be able to accurately measure women’s pregnancy intentions and pre-pregnancy behaviours.

There have been efforts to estimate levels of unplanned pregnancy among populations around the world for almost a century. This has predominantly been undertaken using survey questions (e.g., demographic and health surveys) [23]; however, there has been increased realization, recently, of the complexity of the construct, the need for more sophisticated measurement strategies, incorporating uncertainty, ambivalence, etc., [24,25,26], and the potential role of psychometric methods for measurement in this area. The London Measure of Unplanned Pregnancy (LMUP) has been one such tool proposed, based on the women’s views on unplanned pregnancy, first developed in the United Kingdom using a latent-trait model of measurement based on a conceptual model of pregnancy planning/intention developed from women’s accounts of the circumstances of their pregnancies [8]. The LMUP’s underpinning conceptual model comprises six thematic areas, grouped into three domains: (1) stance (expressed intention, desire for motherhood); (2) context (personal circumstances/timing, partner influences); and (3) behaviour (contraceptive use, pre-conceptual preparations), which informed item development. The LMUP has since been validated in 19 other languages and across 16 low-to-high income countries across all continents [27]. The LMUP has been shown to be more reliable than the DHS pregnancy intention questions [28], but as with all psychometric measures, new language versions must be appropriate *to* and evaluated *in* the socio-cultural context. The aims of this research were therefore as follows: (1) translate the LMUP into Italian language and validate it, investigating its psychometric properties; (2) to test the cultural invariance of the LMUP between Italian and UK contexts.

## 2. Materials and Methods

### 2.1. Ethics

The studies involving human participants were reviewed and approved by the Ethics Committee of Pisa University Hospital (CEAVNO ref. 12749, approved on 22 July 2021) and University of Liverpool (ref: IPH/3964, approved on 30 July 2021) research ethics committees. The participants provided their written informed consent to participate in this study. Ethical approval for the original UK LMUP development and evaluation study was granted by the National Health Service London Regional Multicenter Research-Ethics Committee (ref. MREC 98/2/69, approved on 28 July 1998).

### 2.2. Translation Process

After the approval of the LMUP working group, the translation followed accepted international standards [29,30,31]. The LMUP [32] was translated from English to Italian with the collaboration of three expert translators, professionals in the field of perinatal mental health who worked independently. The Italian versions were back-translated into English by two expert psychologist translators not involved in the previous phase, and who were not familiar with the scale. Finally, the expert group, which included two experts in the LMUP (including the developer of the LMUP) checked the translation against the original without making cultural adaptations. The final Italian version of LMUP is available on the website [33].

### 2.3. Data Collection Procedure

For this study, women with an infant up to 6 months of age were recruited through online advertisements between March and April 2022. These advertisements were disseminated on social media and parenting forums via a link to the Qualtrics 2022 software, which remained active until the end of the follow-up phase of the entire study. Information on the first page explained the aims of the study, that participation was voluntary and that no reward would be given. Informed consent was obtained from all participants. To maintain anonymity, the survey programme included a unique ID linked to each response. The database was stored on a secure server, and access to the information was limited to the research team members. To be included in the study, women had to be aged over 18 years and have a good knowledge of the Italian language. Exclusion criteria were cognitive impairment and psychiatric disorders.

### 2.4. Measures

All participants completed a self-reported questionnaire consisting of personal data information and psychometric measures. The latter included the LMUP to assess the circumstances of pregnancy; the Edinburgh Postnatal Depression Scale (EPDS) [34] to measure the level of depressive symptoms; the Generalized Anxiety Disorder-7 (GAD-7) [35] and the Postpartum Specific Anxiety Scale (PSAS) [36] to assess the anxious symptoms; the Baby Care Questionnaire Version 2 (BCQ-2) [37] to measure the parental beliefs about the baby care practices; and the Postpartum Bonding Questionnaire (PBQ) [38] to assess the quality of the parent–baby bond. The questionnaire requests responses for every question, ensuring that respondents answer before proceeding. Therefore, there were no missing data.

#### 2.4.1. Demographics

Demographic data were collected through a questionnaire aimed at investigating both personal and infant information. This included age, race educational level, occupational status, marital status, and baby birth order.

#### 2.4.2. The London Measure of Unplanned Pregnancy

The London Measure of Unplanned Pregnancy (LMUP) is a self-administered tool consisting of six questions through which women report the circumstances of their recent pregnancy (contraceptive use, timing of becoming a mother, expressed intention, desire for a baby, discussions with partner, and pre-conception preparation). Each item has a score of 0, 1, or 2. These scores are summed to create an ordinal variable on a scale of 0–12, with each increase in score reflecting an increase in pregnancy intention [7,20]. The main advantage of the LMUP is to allow a range of positions in relation to pregnancy planning to be expressed, including ambivalence. For the purposes of interpretation [8,21], the LMUP scores may be understood as unplanned (0–3 points), ambivalent (4–9 points), or planned (10–12 points). The original LMUP had high reliability (Cronbach’s α = 0.92; test–retest reliability = 0.97) and high validity of face, content, and construct [7], with subsequent translations also performing well. Moreover, it is suitable for use with any pregnancy, regardless of outcome.

#### 2.4.3. The Edinburgh Postnatal Depression Scale

The Italian version [39] of the Edinburgh Postnatal Depression Scale (EPDS) [34] was used to investigate depressive symptoms. EPDS is a self-report instrument consisting of 10 items. Individuals are required to indicate how they felt in the past week (e.g., “I have blamed myself unnecessarily when things went wrong”). Responses are given on a 4-point Likert scale (from 0 to 3). The total EPDS score ranges from 0 to 30, with higher scores indicating more severe depressive symptoms. Reliability for the current sample ωT = 0.90.

#### 2.4.4. The Generalized Anxiety Disorder-7

The Italian version [40] of the Generalized Anxiety Disorder-7 (GAD-7) [35] was used to investigate the presence of generalized anxiety symptoms. The GAD-7 is a self-report questionnaire composed of 7 items. Subjects are required to indicate whether they have suffered from anxiety in the last two weeks (e.g., “feeling nervous, anxious, or on edge”). Responses are given on a 4-point Likert scale (from 0 to 3). The total score ranges from 0 to 21, with higher scores indicating more severe anxious symptoms. Reliability for the current sample ωT = 0.90.

#### 2.4.5. Postpartum Specific Anxiety Scale

The Italian version [41] of the Postpartum Specific Anxiety Scale (PSAS) [36] was used to assess the specific postpartum anxiety. The PSAS-IT is a self-report questionnaire consisting of 51 items that assess the frequency of specific anxiety symptoms in the past week during the postpartum period (e.g., “I have worried about my baby’s weight”). Responses are rated on 4-point Likert scale from 1 (never) to 4 (almost always). The total score on the PSAS ranges from 51 to 204, with higher scores indicating a higher level of anxiety. Reliability for the current sample ωT = 0.94.

#### 2.4.6. Postpartum Bonding Questionnaire

The Italian version [42] of the Postpartum Bonding Questionnaire (PBQ) [38] is used to assess the quality of the parent–baby bond. It consists of 25 items assessing the attitude that a parent may exhibit towards their child and that may negatively affect the parent–child bond. These attitudes are impaired bonding, rejection and pathological anger, anxiety for the child and care, and imminent abuse or risk of abuse. Respondents are asked to indicate how often they experience the feelings expressed in each item (e.g., “I feel distant from my baby”) using a 6-point Likert scale ranging from 0 (never) to 5 (always). The Italian version of PBQ used in this study consists of three subscales (‘annoyance and anger’, ‘detachment and rejection’ and ‘anxiety about infant care’) and a total score ranging from 0 to 125. The items describing a positive bonding are scored in the opposite direction. Higher total scores indicate a more impaired mother–child bond. Reliability for the subscales in the current sample are Impaired bonding ωT = 0.90, Rejection Pathological anger ωT = 0.87, Infant-focused anxiety ωT = 0.61, and Incipient abuse r_sb_ = 0.71 (notably, the latter is split half reliability as this subscale has two items).

#### 2.4.7. Baby Care Questionnaire Version 2

The Italian version [43] of Baby Care Questionnaire Version 2 (BCQ-2) [37] was used to measure parental beliefs about sleeping, eating, and consoling the baby. The BCQ-2 contains 30 items asking parents (a) to rate their agreement (1 = strongly disagree; 4 = strongly agree) and (b) to describe their practices using checklists and quantitative questions (such as estimated duration) (example of item is “My babies slept in my bed”). The BCQ-2 allows measurement of the structure of caregiving (the extent to which parents endorse routine and regularity in childcare) and attunement (the extent to which parents endorse close physical contact and rely on the child’s signals). Higher scores indicate higher structure and attunement.

### 2.5. Data Analysis: Evaluating Psychometric Properties of the LMUP-IT and Hypotheses

Analyses were based on the Classical Test Theory, which underpins the original LMUP, carried out using the “psych” and “Lavaan” packages in R 4.5.0, SPSS 30, and Stata18.

#### 2.5.1. Targeting

We examined item category endorsement values for insight into item discrimination, and we assessed targeting by examining the distribution of total LMUP scores to see if the full range of scores was achieved and to assess the shape of the distribution. A full range of scores was our ideal, but we deemed the presence of all but the lowest scores as acceptable targeting, in keeping with comparable samples in other high-income, low-fertility countries, e.g., Belgium [8], Australia [44]. We expected to see a negatively skewed distribution, i.e., a large number of high LMUP scores, with a skew towards lower scores.

#### 2.5.2. Reliability

As in previous LMUP evaluations, we examined inter-item correlations to check they were all positive, and item–rest correlations were considered acceptable if they were above 0.2 [45]. Internal consistency was evaluated with Cronbach’s alpha, using the standard cut off of 0.7 [46]. Internal reliability was also assessed with McDonald’s omega (ωT), as this is now considered more robust than alpha [47,48], with a cut off of 0.7 for McDonald’s omega.

#### 2.5.3. Construct Validity

According to the literature and COSMIN convention [30,49], we assessed the construct validity of the LMUP-IT by using a range of methods. We assessed structural validity using Confirmatory Factor Analysis (CFA). Due to the three-level ordinal nature of the data and the extreme skew in responses, the CFA was fit with a diagonally weighted least squared estimator (we also confirmed this fit using maximum likelihood with robust standard errors). A range of fit indices were produced to test the factor structure. The baseline comparison Comparative Fit Index (CFI) was deemed acceptable at  >0.90 and good at  >0.95. The standardized root mean residual (SRMR) absolute fit index was used, with values under 0.08 being indicative of good fit. Finally, the RMSEA parsimony adjusted measure is reported, with values  < 0.06 indicating good fit and values  > 0.06 but  <0.08 being acceptable. Given that in many previous evaluations of the LMUP the structural validity of the scale was assessed using principal components analysis (PCA), we present the PCA findings, a legacy analysis, in the Supplementary Materials (Tables S1 and S2 and Figures S1 and S2) to allow direct comparison. In these, the unidimensionality of the scale was confirmed if all items loaded onto one component with an Eigenvalue greater than 1.

To formally assess the cross-cultural validity of the scale in Italian, we conducted measurement invariance testing between the Italian data and a UK sample. To do this, we matched the Italian data with a UK sample [8]. To ensure that invariance/non-invariance could be attributed to country, we matched the samples based on age and partnership (women living with partner and living with husband). We would not have been able to accurately match the samples based on the absence of a partner (single/separated) because the most individuals in the Italian sample were in a couple relationship (99.6%). Therefore, the unpartnered participants were excluded by both (Italian and UK sample). This gave two matched samples consisting of 279 participants in each. Both samples had a mean age of 33.29 (±3.7) years, and within each sample 213 lived with their husband and 66 lived with their partner. Further, 279 participants in each group gave just under 46 participants per item, which is in excess of recommended N-per item cut offs, and in excess of simulation-based recommendations given the factor structure [50].

Configural invariance (factor structure holding across the countries) was tested through fitting the factor structure with country as a grouping variable. This model was assessed using the fit indices described above for the CFA. The configural model was then compared to the metric invariance model, where factor loadings are fixed across groups. This tests the extent to which items contribute to the factor in a similar way across the countries.

Metric invariance was assessed using CFI (∆CFI) < 0.01, RMSEA differences (∆RMSEA) < 0.015, and SRMR differences (∆SRMR) < 0.03 as the cut-offs. Next, the metric invariance model was compared to a scalar invariance model where intercepts are assumed to be equal across countries (along with loadings). This demonstrates of the means of the scale can be validly compared across countries. This was performed in the same way as scalar invariance for metric invariance, except the cut off for ∆SRMR was <0.015.

Finally, strict invariance in which residuals are also assumed to be consistent across countries was tested, which demonstrates if the item residual variance is consistent across groups. The strict invariance model was compared to the metric invariance model (cut-offs for fit indices’ differences were the same as in the previous model comparison) [51].

We used ‘known groups’ hypothesis testing to assess the relationship of the LMUP scores with parity. Given that most women in Italy (60%) express a desire for two children [52], we tested the hypothesis that births of the third child or higher would have lower LMUP scores than lower birth orders.

### 2.6. Exploratory Analysis of LMUP with Other Measures

We explored the relationship of the LMUP-IT with other measures. In line with the literature [17,18,19], we hypothesized that higher LMUP scores (indicating planned pregnancy) are negatively associated with psychological distress (in terms of EPDS, GAD and PSAS) and negative bonding (PBQ) and positively with parental caring (BCQ).

## 3. Results

### 3.1. Sample Description

Table 1 reports the characteristics of the Italian sample. National comparison data are inserted, derived from the statistics about the birth events in Italy in 2023—data for couple status and baby order are not present in the national dataset [53]. The study sample consisted of 450 women aged between 18 and 48 years (mean age 33.60; SD 4.51), mostly of Italian nationality. Participants reported a high educational level (more than 60% of participants had a university degree or higher) and most of them (90%) were employed. Moreover, 99.5% of participants were in a couple relationship. Finally, 69.3% of participants gave birth to their first child.

### 3.2. Targeting

Endorsements of the item response options are shown in Table 2. The endorsement frequency was over 90% on item 1 (contraception, category 2) and item 2 (timing, category 2).

**Figure 1:**
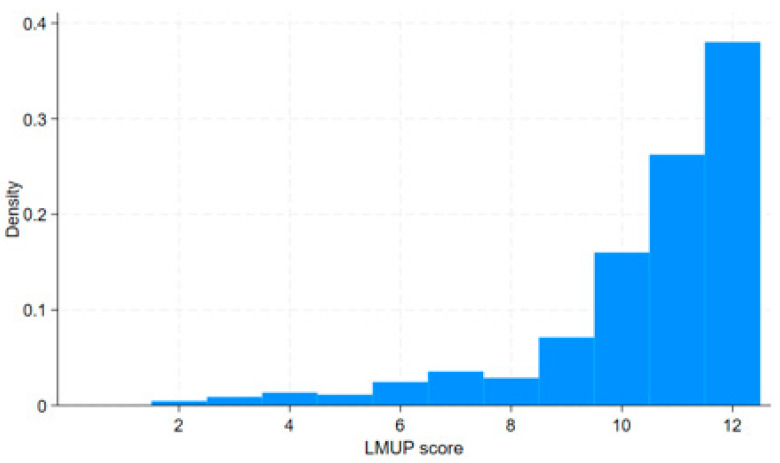
Histogram of LMUP scores.

Total LMUP scores ranged from 2 to 12, with a negatively skewed distribution (Figure 1), a median score of 11 (inter-quartile range 10–12), and mean score of 10.5 (SD 2.0). In total, 6 women (1.3%) scored 0–3 (unplanned), 83 (18.4%) scored 4–9 (ambivalent), and 361 (80.2%) scored 10–12 (planned).

### 3.3. Reliability

All inter-item correlations were positive, and all item–rest correlations were >0.2 (item 1—0.25, item 2—0.48, item 3—0.72, item 4—0.62, item 5—0.58, item 6—0.38). Cronbach’s alpha = 0.76, and ωT = 0.81.

### 3.4. Construct Validity

#### 3.4.1. CFA in Italian Sample

**Figure 2:**
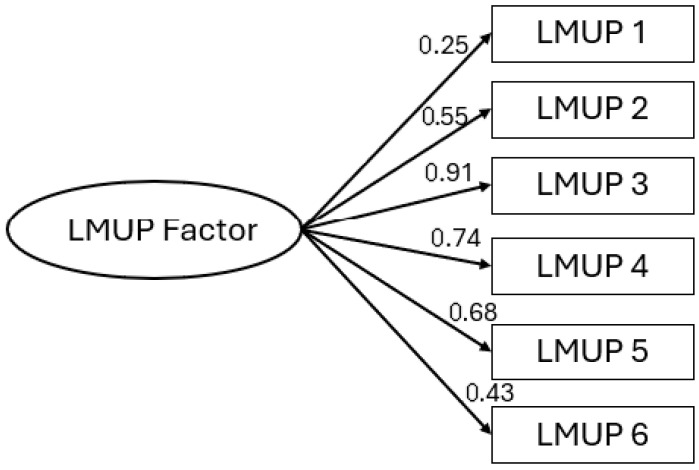
Standardized factor loadings for the LMUP.

The structure was found to be a good fit on all indices with the DWLS estimator (χ^2^(9) = 2.55, RMSEA < 0.001, CFI = 0.99, SRMR = 0.025) and with the MLR estimator also finding a good fit (χ^2^(9) = 21.89, RMSEA = 0.056, CFI = 0.98, SRMR = 0.038). All items had a significant loading on the factor (*p*-values < 0.001), and standardized factor loadings can be seen in Figure 2.

#### 3.4.2. Cross-Cultural Validity—Measurement Invariance Testing

The configural invariance model was a good fit (χ^2^(18) = 3.45, RMSEA < 0.001, CFI > 0.99, TLI > 0.99, SRMR = 0.026) on all indices; see Table 3. The LMUP also demonstrated metric invariance on two measures ∆CFI < 0.001, ∆RMSEA < 0.001, but was above the cut off for the ∆SRMR < 0.038 (allowing LMUP item 4 to vary achieves metric invariance on this measure too). There was scalar invariance, ∆CFI < 0.001, ∆RMSEA < 0.001, ∆SRMR < 0.004, and strict invariance, ∆CFI < 0.001, ∆RMSEA < 0.001, ∆SRMR < 0.012.

Fit indices for each model are as follows: metric (χ^2^(23) = 16.58, RMSEA < 0.001, CFI > 0.99, TLI > 0.99, SRMR = 0.064), scalar (χ^2^(28) = 21.619, RMSEA < 0.001, CFI > 0.99, TLI > 0.99, SRMR = 0.066), and strict (χ^2^(18) = 3.45, RMSEA = 0.001, CFI > 0.99, TLI > 0.99, SRMR = 0.078).

#### 3.4.3. Hypothesis Testing with ‘Known Groups’

The relationship of the LMUP scores to birth order demonstrated a median LMUP score of 11 (IQR 10–12) for first birth, 11 (IQR 10–12) for second birth, and 10 (IQR 8–11) for third-plus birth, *p* = 0.002 (Figure S3).

### 3.5. Exploratory Analysis of LMUP with Other Measures

Scores on the LMUP showed significant positive correlations with PSAS total scores, BCQ structure, and negative associations with EPDS, GAD-7, impaired bonding, and rejection pathological anger (PBQ). There was no significant association between LMUP scores and incipient abuse and BCQ structure; see Table 4.

## 4. Discussion

### 4.1. Summary of Main Findings

Overall, the results demonstrated that the Italian version of LMUP exhibited good psychometric properties according to internationally accepted criteria, in keeping with previous international LMUP evaluations and in line with the original psychometric model, confirming its robustness as a measure of pregnancy planning. Specifically, the LMUP-IT proved acceptable targeting, good internal consistency (reliability), and good construct validity in terms of hypothesis testing and structural validity (demonstrating unidimensionality). Further, this evaluation was the first LMUP evaluation to test cross-cultural structural validity (construct validity) via measurement invariance testing, comparing Italy with the UK. The results provide important support for the validity of the Italian version of the LMUP, indicating the measurement model is comparable across countries, at least between Italy and the UK.

The level of pregnancy planning was high in this study, likely due to the high number of married, partnered, and older mothers in the sample. (The high level of pregnancy planning in this study, e.g., 80% with an LMUP score > 9, is, however, in keeping with similar samples from other high-income, low-fertility countries, e.g., 73% in the UK, 85% in Belgium, 74% in Australia) [9,44,54]. In terms of the measurement properties of the items in this study, the high level of pregnancy planning meant there was little discrimination in item 1 (contraception), as most women reported not using contraception. Item 1 also had the lowest standardized factor loading in the Confirmatory Factor Analysis, albeit still contributing towards unidimensional measurement. We believe the lower standardized factor loading is likely an artefact of the lack of item discrimination rather than any inherent poor function of the item; we expect that this measurement property of item 1 will improve when reviewed in more diverse samples in future. Issues have previously arisen with item 1 in contexts with low contraceptive prevalence, which have also limited discrimination and allowed lower item–rest correlations and factor loadings [55]; however, these have sometimes been resolved in larger, more diverse samples [56]. The findings of our analyses also provided useful insights beyond psychometric measurement properties. Whilst this sample had a high proportion of planned pregnancies (LMUP score > 9), preparation for pregnancy, such as folic acid use, was comparatively low, with almost a quarter of women reporting taking no action to prepare for pregnancy.

This suggests a possible lack of awareness or cultural variation in the understanding of actions required to improve preconception health and long-term maintenance of a health pregnancy. In fact, in line with a previous study, although preconception visits are efficacious to improve preconception care, only 35.4% of Italian women underwent a preconception visit [57]. The low preparation for pregnancy and limited use of the preconception visits [57] suggest the need to increase the information about the relevance of preconception care to reduce the risk factors for pregnancy.

### 4.2. Interpretation in Light of Published Literature

Our findings are in line with what is known about the widespread use of non-technological contraceptive methods (such as natural methods or coitus interruptus) [3] in Italy, which increase the possibility of becoming pregnant without actively planning the pregnancy. In fact, whereas the active planning of pregnancy (avoiding using contraception) seems to be linked to choosing the right time to have a child, the difficulty in identifying that right time due to economic conditions, working conditions, and lack of childcare services leads women to “let” births happen (using non-technological contraception) [5].

Furthermore, our results showed that the LMUP was negatively associated with post-natal depressive symptoms (EPDS) and generalized anxiety symptoms (GAD-7). These findings suggest that a lack of planning and intention of pregnancy can lead to an increase in anxious and depressive symptoms. The difficulty in actively planning the pregnancy (due to, for instance, difficult economic conditions, unstable working conditions, or workplaces where maternity lead to be discriminated) [5] may lead woman who become mothers in inappropriate timing to show increased anxiety symptoms due to the conflicts between the mothering and working domains. The anticipated gender discrimination in the workplace, which is difficult to self-protect against, along with the traditional gender roles in heterosexual Italian couples (where women are primarily responsible for the household and childcare), may lead women who become pregnant unexpectedly or at an inconvenient time to believe that combining motherhood with a working life is impossible, thereby increasing anxious symptoms in the postpartum period [58].

Despite the negative association between LMUP and generalized anxiety symptoms, however, we found a positive association between LMUP and postpartum anxiety (PSAS-IT). Moreover, the results showed that the LMUP-IT was significantly and positively associated with the measure of the structure of caregiving (BCQ-2-structure). These findings suggest that a higher degree of pregnancy planning seems to be linked to a greater emphasis on routine and regularity in childcare, possibly reflecting a more structured and intentional approach to parenting. In contrast, the LMUP was significantly and negatively associated with the impaired quality of the parent–baby bond (PBQ). The negative correlation with the quality of the parent–baby bond implies that women who plan their pregnancies experience a stronger bond with their child. This may be because planned pregnancies allow for better emotional preparation, facilitating a more secure attachment with the newborn.

It is possible that the difficult social and economic situation may lead to more pregnancy planning, in line with more optimal conditions for childbearing (e.g., education, stable employment, couple stability, homeowner) [6]. However, when these optimal conditions are achieved, mothers may be aware of possible gender discrimination in the workplace, the traditional gender roles, and about the social construction of the myth of maternity characterized by the ideal of the ‘perfect mother’ who is always available and attentive to the child’s needs, and of the belief that the mother is primarily responsible for childrearing (typical of intensive mothering) [59,60]. Thus, the women may aspire to be a superwoman, achieving impossible perfection as a mother, professional, and wife. These expectations and beliefs may lead to unrealistic standards which are associated with maternal postpartum anxiety [61].

### 4.3. Strengths, Limitations, and Future Directions

Despite these promising results, the present study is not without limitations. The first limitation concerns the convenience sample used, which consists primarily of older and well-educated women in a relationship. This is a common consequence of social media recruitment [62]. Therefore, this study should be replicated with those who have low educational attainment and those who are unpartnered, and it should also aim to include individuals who suffer from ‘digital poverty’ (i.e., those who lack internet access or are not familiar with technology), in order to assess a more heterogeneous and representative sample, ideally using probability sampling, to strengthen the obtained results.

As this was a cross-sectional survey, with data collection at only one time point, we were unable to assess the test–retest reliability (stability) of the LMUP. This could be assessed in a future study. Further, after the initial translation and cultural adaptation stage, we were not able to carry out any cognitive interviews to assess the face validity of the LMUP, albeit the subsequent good psychometric performance of the LMUP-IT suggests that it is unlikely that there are any major problems with the face validity. Nonetheless, face validity can be assessed formally in future studies.

As previously stated, we were unable to test the relationship between the LMUP scores and occupational status since the vast majority of participants were employed and could not test the relationship of LMUP scores with partnership status because most of the participants were partnered. This could be linked to the cultural and social Italian context that emphasizes the myth of ‘natural’ motherhood in a traditional heterosexual couple due to the cultural roots of Catholicism [4,5]. Therefore, in Italy, having a partner significantly influences fertility choices and pregnancy intentionality in comparison to countries with more liberal attitudes towards childbirth outside of a relationship, where being partnered has less of an influence on pregnancy planning [63,64]. Moreover, due to these considerations, single women do not have access to assisted reproduction treatments in Italy. This causes discrimination amongst single people who are excluded by law from the right to achieve pregnant alone [5].

Further, collecting data after childbirth may constitute a selection bias for this study, and certainly women who decided to terminate their pregnancy (including those who may have done so due to the unplanned nature of it) were excluded from the broader study (which otherwise focused on the postpartum mental health of mothers of live infants). As a result of this sample, we did not capture the full range of LMUP scores, missing the women with the most unplanned pregnancies (scores 0 and 1). It is also possible there may be a slight change in the reporting of pregnancy planning after the birth of the child. In this regard, previous studies, such as those conducted in Malawi [55], have shown a small change in the reporting of pregnancy planning from pregnancy to the postpartum period, whilst data from the UK suggest that this shift is relatively stable [8]. Future studies should employ a longitudinal research design to capture any changes between the pre- and post-natal periods. In this context, it could be relevant to capture the intention at the beginning of pregnancy.

Finally, we only used self-reported measures, and it would be valuable to confirm the results using other data collection methods, such as interviews or implicit measures, to overcome potential defensive or self-representational biases.

## 5. Conclusions

The Italian version of the LMUP proves to be a valid tool for providing essential information in the context of pregnancy intention. Specifically, it can contribute to awareness campaigns that promote healthy preconception behaviours and informed contraceptive use, helping individuals to make pregnancy a conscious choice and reducing the psychological distress associated with unplanned pregnancies. By understanding the level of planning behind a pregnancy, interventions can be more effectively tailored to support women’s mental health during the transition to motherhood, further emphasizing the importance of addressing the psychological aspects of reproductive health.

## Figures and Tables

**Table 1:** Characteristics of study sample and comparison with national Italian data.

	*n* (%)	National Data
**Nationality**		
Italian	437 (97.1%)	79.9%
Others	13 (2.9%)	20.1%
**Educational level**		
Secondary school	20 (4.4%)	22.0%
High school	158 (35.1%)	42.4%
University degree	272 (60.4%)	35.6%
**Occupational status**		
Employed	405 (90%)	60.1%
Unemployed	21 (4.7%)	14.2%
Housewife	24 (5.3%)	23.7%
**Marital status**		
Married	248 (55.1%)	56.4%
Maiden	201 (44.6%)	41.7%
Separated	1 (0.2%)	1.9%
**Couple status**		
In couple (married and/or coresident)	448 (99.5)	-
Single	2 (0.5)	-
**Baby birth order**		
1°	312 (69.3%)	-
2°	112 (24.9%)	-
3°	20 (4.4%)	-
4° or more	6 (1.3%)	-

**Table 2:** Item category endorsement frequencies.

Items and Response Categories	*n* (%)
**Item 1: Contraception**	
0—always using contraception	8 (1.8)
1—using sometimes or failed at least once	15 (3.3)
2—not using contraception	427 (94.9)
**Item 2: Timing**	
0—wrong time	5 (1.1)
1—ok, but not quite right time	39 (8.7)
2—right time	406 (90.2)
**Item 3: Intention**	
0—did not intend to get pregnant	25 (2.6)
1—intentions kept changing	48 (10.7)
2—intended to get pregnant	377 (83.8)
**Item 4: Desire for a baby**	
0—did not want to have a baby	10 (2.2)
1—mixed feelings about having a baby	85 (18.9)
2—wanted to have a baby	355 (78.9)
**Item 5: Partner discussion**	
0—Never discussed having children or solo pregnancy	3 (0.7)
1—discussed but not agreed to get pregnant	78 (17.3)
2—Agreed to get pregnant	355 (82.0)
**Item 6: Preconceptual preparations**	
0—no actions	102 (22.7)
1—1 action	124 (27.6)
2—2 or more actions	224 (49.8)

**Table 3:** Tabulated measurement invariance change statistics.

Invariance	∆CFI	∆RMSEA	∆SRMR
Metric	<0.001	<0.001	<0.038 *
Scalar	<0.001	<0.001	<0.004
Strict	<0.001	<0.001	<0.012

* SRMR < 0.004 when allowed item 5 to vary.

**Table 4:** Pearson’s correlations between the LMUP and other measures.

Measure	*r*, (*df*), *p*
PSAS Total	0.10, (447), *p* = 0.034
EPDS Total	−0.12, (439), *p* = 0.011
GAD Total	−0.14, (430), *p* = 0.003
PBQ Impaired Bonding	−0.22, (427), *p* < 0.001
PBQ Rejection Pathological anger	−0.19, (427), *p* < 0.001
PBQ Infant-focused Anxiety	−0.22, (427), *p* < 0.001
PBQ Incipient Abuse	−0.08, (427), *p* = 0.090
BCQ Attunement	0.03, (322), *p* = 0.562
BCQ Structure	0.13, (322), *p* = 0.017

## Data Availability

The raw data supporting the conclusions of this article will be made available by the authors, without undue reservation, upon reasonable request to the corresponding author, but are not readily available or stored in a repository as they rely on clinical data from both countries and therefore privacy and confidentiality are paramount.

## Supplementary Materials

The following supporting information can be downloaded at https://www.mdpi.com/article/10.3390/healthcare13162052/s1, Table S1: Principal Component Analysis (PCA) based on Pearson correlations; Table S2: Principal Component Analysis (PCA) based on polychoric correlations; Figure S1: Scree plot after Principal Component Analysis (PCA) based on Pearson correlations; Figure S2: Scree plot after Principal Component Analysis (PCA) based on polychoric correlations; Figure S3: Boxplot of LMUP scores by birth order.
