# Supplementary material for: Psychometric Evaluation of the Validity and Reliability of the Italian Version of the London Measure of Unplanned Pregnancy Amongst Postnatal Women
Source: Healthcare (Basel). 2025 Aug 20;13(16):2052. doi: 10.3390/healthcare13162052 (PMC12386161; doi:10.3390/healthcare13162052)
Supplement: Supplementary file 1 [file healthcare-13-02052-s001.zip › healthcare-3596938-supplementary.pdf]

Table S1. Principal Component Analysis (PCA) based on Pearson correlations

**Items**	**Component loadings**	**Eigenvalue**
1	0.36	Eigenvalue=2.8<br><br>(One Eigenvalue >1 explaining 48% of variance)
2	0.68	
3	0.88	
4	0.82	
5	0.75	
6	0.54	
These PCA findings are consistent with the PCA analyses presented in previous LMUP evaluations; PCA based on Pearson correlations.		

Table S2. Principal Component Analysis (PCA) based on polychoric correlations

Items	Component loadings	Eigenvalue
1	0.58	Eigenvalue=3.9<br><br>(One Eigenvalue >1 explaining 64% of variance)
2	0.79	
3	0.96	
4	0.88	
5	0.87	
6	0.66	

These PCA findings, based on polychoric correlations, are now considered more appropriate for use with ordinal data, such as LMUP scores.

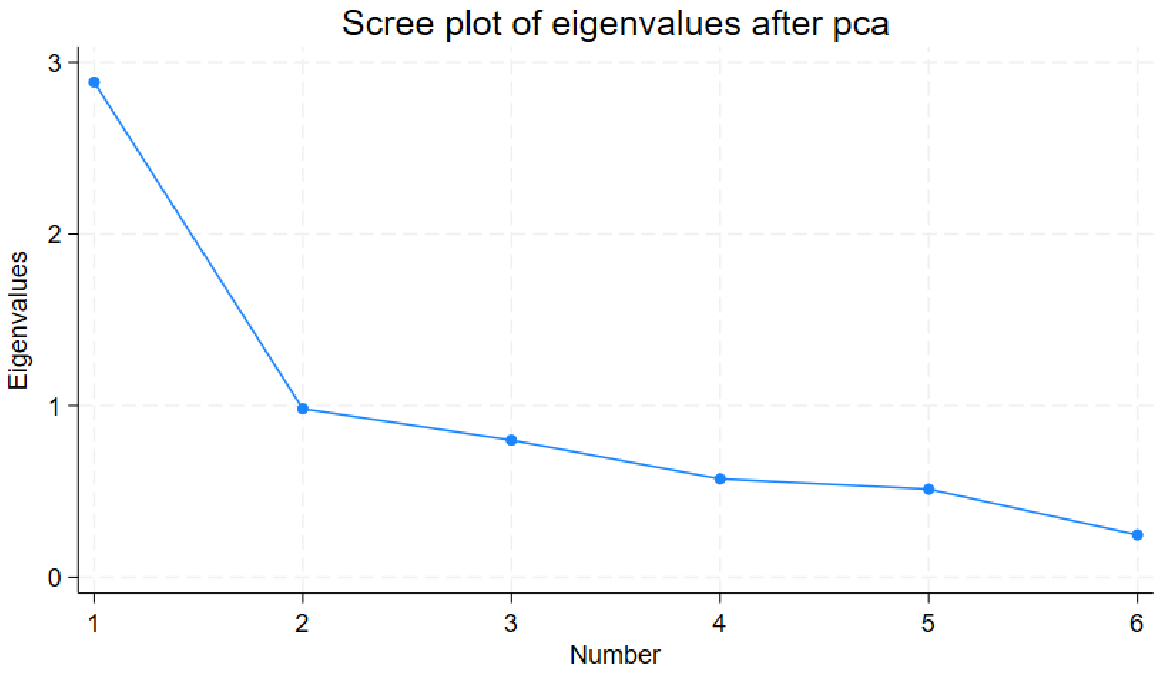

Figure S1. Scree plot after Principal Component Analysis (PCA) based on Pearson correlations

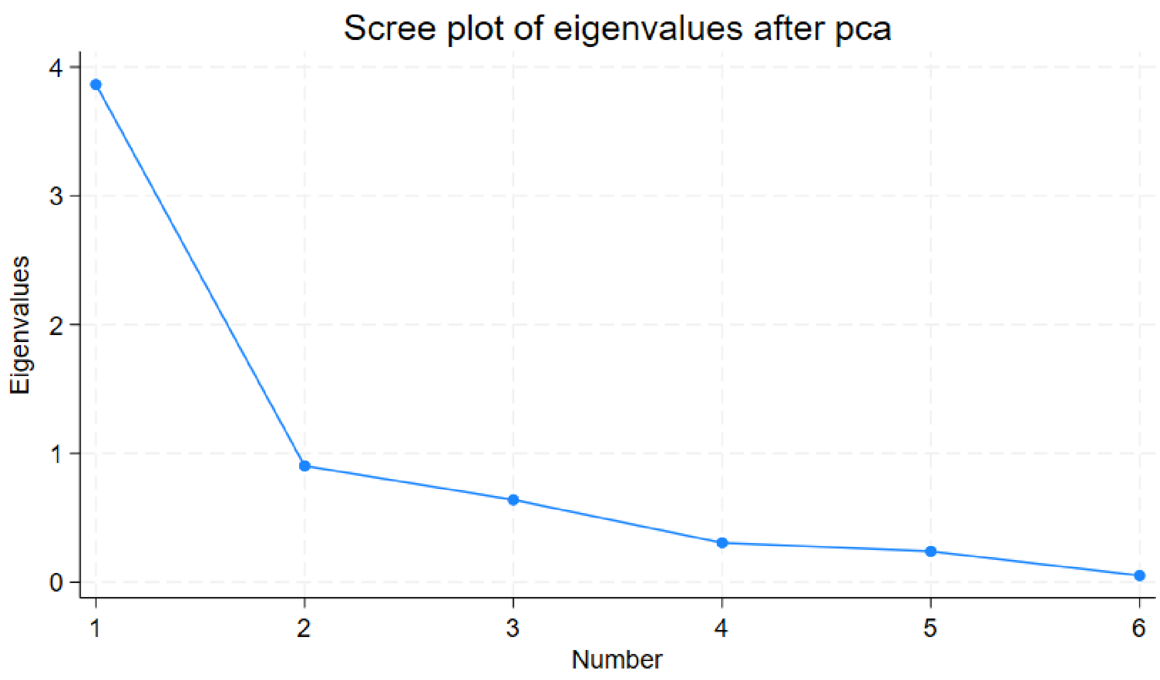

Figure S2. Scree plot after Principal Component Analysis (PCA) based on polychoric correlations

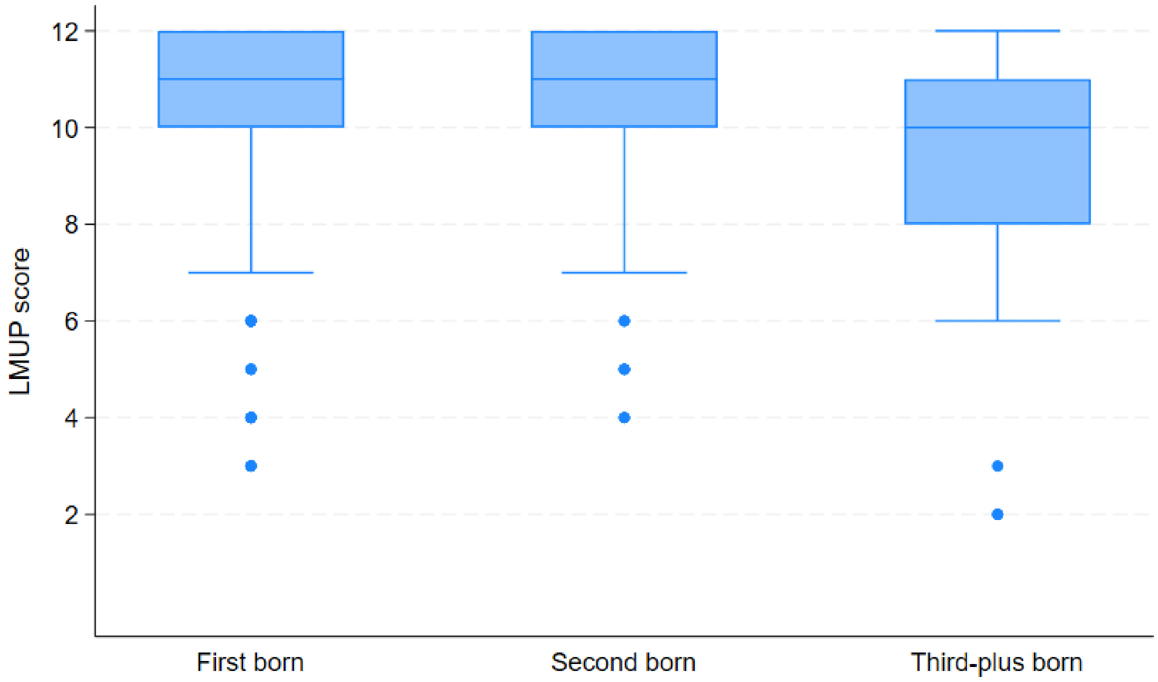

Figure S3. Boxplot of LMUP scores by birth order
